# Barriers for multiparous women to using long-term contraceptive methods in Southeast Asia: case study in Philippines and Indonesia

**DOI:** 10.1186/s12889-022-13844-z

**Published:** 2022-07-27

**Authors:** Agung Dwi Laksono, Nikmatur Rohmah, Hario Megatsari

**Affiliations:** 1 National Research and Innovation Agency, The Republic of Indonesia Jakarta, Indonesia; 2 Faculty of Health Science, Muhammadiyah University of Jember, East Java, Indonesia; 3 Faculty of Public Health, University of Airlangga, Surabaya, Indonesia

**Keywords:** Multiparous women, Parity, Long-term contraceptive methods, Contraception, Public health

## Abstract

**Background:**

Multiparous women are supposed to be able to end their reproductive cycle to decrease population growth. This study aimed to analyze barriers for multiparous women to use long-term contraceptive methods (LTCM) in the Philippines and Indonesia.

**Methods:**

The study population was women aged 15–49 years old who have given birth to a live baby > 1 in the Philippines and Indonesia. The weighted sample size was 12,085 Philippines women and 25,543 Indonesian women. To identify variables associated with the use of LTCM, we analyzed place of residence, age group, education level, marital status, employment status, and wealth status. The final step employed multinomial logistic regression.

**Results:**

In both countries, the results showed that variables associated with non-user LTCM were younger women, living in rural areas with poor education. Women without partner and unemployed had higher probability to not use LTCM. Finally, low wealth status had a higher probability than the richest multiparous to not use LTCM.

**Conclusion:**

The study concluded that there were six barriers for multiparous women to use LTCM in the Philippines and Indonesia. The six obstacles were living in rural areas, being younger, poor education, single, unemployed, and low wealth.

## Introduction

Population growth in Southeast Asia has outpaced growth in the U.S., U.K., and China in the last 5 years. Meanwhile, the population growth rate in Southeast Asia is 5.8%. Philippines population growth of 8.2% is the highest growth compared to other Southeast Asian countries. Indonesia’s population grows by 5.9%, just below Malaysia’s at 7.9% [1].

Southeast Asia’s current population is 672,082,017, equivalent to 8.58% of the world’s total population. Southeast Asia is ranked number 3 in the Asian region. The population density in Southeast Asia is 154 per km2, and 50% reside in urban areas. The mean age of the Southeast Asian population was 30.2 years. Based on population, Indonesia is ranked first in the Southeast Asia region and fourth globally, with 273,523,615 people. The Philippines is in second place in the Southeast Asia region and 13 globally, with 109,581,078 people. The rapid population growth in Indonesia and the Philippines indicates that the number of pregnancies that reach proper delivery in both countries is high [2, 3].

The number of pregnancies that achieve a viable delivery referred to as parity [4]. Women who have given birth more than two times are called multiparous and have given birth ≥ four times called grand multiparous. The more often a woman gives birth, the more it has the potential to increase population growth. The crude birth rates in the Philippines and Indonesia are ranked 4th and 5th in the Southeast Asia region, namely 20.2 and 17.7 live births per 1000 population [5]. The family size in the Philippines also allows a woman to give birth more than two times. The Philippines’ average ideal family size is 2.7 children for all women and 3.0 children for currently married women [6].

The high proportions of multiparous and grand multiparous cause population growth to occur rapidly. The distribution of currently married women in Indonesia is 25.57% multiparous and 4.27% grand multiparous. The remaining 62.58% were primiparous, and 7.57% never gave birth based on parity [7]. In some countries, the proportion of multiparous shows a higher number than primiparous. Among them are Nigeria, Uganda, Northwestern Ethiopia, and North Carolina [8–11]. This situation is different when we compare it to China. The proportion of multiparous women is 15.5%, primiparous 29.1, and 55.4% childless [12].

The utilization of LTCM is related to many factors, several studies showed that facts [13–15]. One of the factors is related to pregnancy, both planned and unplanned pregnancies. For example study conducted by Pasundani in 2020 [15] in Indonesia, age, education, parity, and information are all variables that have been linked to the usage of LTCM. Other study revealed the same messages that unplanned pregnancy was also factors for women utilize the LTCM [16].

Meanwhile, women who give birth more than two times have the risk of various complications of pregnancy and childbirth and even death. The risk of death from maternal causes is related to pregnancy risk and complications during birth [17]. The three causes of maternal death in the Philippines include complications of delivery, pregnancy-related hypertension, and postpartum hemorrhage [18]. Multiparous women have less time to recover their reproductive organs and less time to rest. Multiparous has an increasing burden as the number of children born. In general, the history of miscarriage and gestational diabetes was higher in the grand multiparous [19].

Previous studies found a significant association between grand multiparous and pregnancy outcome. Among them include cesarean section, fetal macrosomia, diabetes mellitus, hypertension in pregnancy caused [20]. Multiparity is still a threat to women’s health problems. This threat is mainly related to the various complications that can occur during pregnancy and childbirth. Efforts to minimize this threat are to carry out pregnancy and birth control.

Long-term contraceptive methods (LTCM) are the most effective method for controlling pregnancy and birth [21]. Including the LTCM are an intrauterine device (IUD), female sterilization, and implant/Norplant. LTCM can prevent unwanted pregnancy up to 20 times better than pills and vaginal rings [22]. LTCM is a form of birth control that is very effective and has the lowest maintenance costs (most economical) [9, 23]. Unfortunately, women attracted to LTCM but did not use it are more likely to be multiparous women [11]. Although LTCM is proven to reduce pregnancy effectively and is effective regardless of user compliance, its absorption is still low [24, 25].

A previous study in seven countries found that the widespread use of LTCM was still low, namely 16% [26]. IUDs and implants’ use is still low, 1.8 and 10.4%, respectively [9]. Policymakers need to know more information about the various challenges in women’s access to use LTCM. Moreover, we need to investigate this further. This study analyzes the obstacles for multiparous women to use LTCM in the Philippines and Indonesia based on the background description.

## Materials and methods

### Availability of data and materials

The author is unable to share the data publicly because a third party and the ICF, who own it, do not have permit to do so. The author requested The 2017 IDHS and 2017 PDHS data set to the ICF (data set of childbearing age women) and the ICF gave the access to confidential data through https://dhsprogram.com.

### Data source

The report used secondary data as research materials from the 2017 Philippines Demographic and Health Survey (PDHS) and 2017 Indonesian Demographic and Health Survey (IDHS). The PDHS and IDHS are part of the Inner City Fund’s (ICF) Global Demographic and Health Survey (DHS) survey collection. The sample population was women aged 15–49 years who had given birth in Indonesia to a live baby of over 1 (multiparous). The sampling procedure used stratification and multi-stage random sampling. The method yielded a weighted sample size of 12,085 Filipino women and 25,543 Indonesian women. The study period was from July 24th – September 30th 2017.

### Data analysis

The dependent variable in this study was the use of long-term contraceptive methods (LTCM). In this study, LTCM is a modern contraceptive type consisting of IUD, female sterilization, and implant/Norplant. The study divides the use of LTCM into 2 categories, namely “Yes” and “No.”

There were six independent variables involved in the analysis of this study. The six variables are a type of place of residence, age group, education level, marital status, employment status, and wealth status. The residence type consists of 2 categories, namely “urban” and “rural.” The urban-rural criteria refer to Statistics Indonesia. Age group is divided into 7 categories in 5 years, namely “15–19”, “20–24”, “25–29”, “30–34”, “35–39”, and “40–45”. Education level is the respondent’s recognition of the last diploma they have. Education level is divided into 4 categories, namely “no education”, “primary”, “secondary”, and “higher”. The study also divided marital status into two categories: “single” and “married/living with a partner.” The study divided into two categories for employment status, namely “unemployed” and “employed.”

The study determined wealth status based on the quintile of wealth owned by a household. Households were scored based on the number and type of items they have, from televisions to bicycles or cars, and housing characteristics, such as drinking water sources, toilet facilities, and primary building materials for the house’s floor. This score calculated using principal component analysis. National wealth quintiles were arranged based on household scores for each person in the household and then divided by the distribution into the same five categories, accounting for 20% of the population [27].

In the first step, the authors conducted a collinearity test. The action is to ensure no multicollinearity between the independent variables. Furthermore, the study carried out a bivariate test between the dependent and independent variables using the Chi-Square test. The study used the binary logistic regression to analyze barriers for multiparous women to use LTCM in Indonesia and Philippines in the final stage, and we used the significancy *p* < 0.05. We used 95% confidence interval in the binary logistic regression. All stage statistical analyzes carried out using SPSS 22 software.

### Ethical statement

The 2017 PDHS and the 2017 IDHS have received ethics approval from the national ethics commission at the Ministry of Health, Philippines, and Indonesia. The authors deleted the respondents’ identities from the dataset. Besides, respondents have provided written approval for their involvement in the study. The author has obtained permission to use Inner City Fund International’s data through its website: https://dhsprogram.com/data/new-user-registration.cfm.

## Results

Table 1 showed the results of the co-linearity test. The analysis results displayed the tolerance value for all independent variables was > 0.10, and the variance inflation factor (VIF) value for all independent variables was < 10.00. The results of this analysis informed that there were no symptoms of multicollinearity between the independent variables.

**Table 1:** The result of multicollinearity test

Independent Variables	Collinearity Statistics			
	Philippines (***n*** = 12,085)		Indonesia (***n*** = 25,543)	
	Tolerance	VIF	Tolerance	VIF
Type of place of residence	0.892	1.121	0.799	1.252
Age group	0.900	1.111	0.903	1.107
Education Level	0.710	1.408	0.745	1.343
Marital status	0.982	1.018	0.979	1.021
Employment status	0.928	1.077	0.955	1.047
Wealth status	0.629	1.590	0.659	1.517

Dependent variable: The use of LTCM

### Descriptive analysis

Table 2 showed descriptive statistics of multiparous women in the Philippines and Indonesia. Based on the type of place of residence, Philippines multiparous women who live in rural areas dominated the two categories of the use of LTCM. Meanwhile, multiparous Indonesian women who use LTCM were dominated by those who live in urban areas. Meanwhile, multiparous Indonesian women who do not use LTCM were dominated by those who live in rural areas.

**Table 2:** Descriptive statistics of multiparous women in Philippines (n = 12,085) and Indonesia (*n* = 25,543)

Variables	The use of LTCM					
	Philippines			Indonesia		
	Yes	No	P	Yes	No	P
**Type of place of residence**			***0.000			***0.000
• Urban	46.2%	43.9%		53.2%	47.8%	
• Rural	53.8%	56.1%		46.8%	52.2%	
**Age group**			***0.000			***0.000
• 15–19	0.3%	0.5%		0.0%	0.1%	
• 20–24	4.7%	6.2%		1.3%	2.1%	
• 25–29	10.4%	15.0%		7.3%	9.2%	
• 30–34	16.1%	18.9%		16.5%	19.3%	
• 35–39	21.4%	21.4%		26.9%	24.6%	
• 40–44	25.6%	18.6%		28.0%	22.3%	
• 45–49	21.6%	19.5%		19.9%	22.4%	
**Education Level**			***0.000			***0.000
• No education	0.6%	1.4%		1.1%	2.6%	
• Primary	19.3%	21.3%		33.4%	40.9%	
• Secondary	52.6%	48.0%		48.8%	47.6%	
• Higher	27.5%	29.4%		16.8%	8.9%	
**Marital status**			***0.000			***0.000
• Single	5.4%	8.3%		1.5%	6.1%	
• Married/Living with partner	94.6%	91.7%		98.5%	93.9%	
**Employment status**			***0.000			***0.000
• Unemployed	45.0%	48.5%		39.2%	41.5%	
• Employed	55.0%	51.5%		60.8%	58.5%	
**Wealth status**			***0.000			***0.000
• Poorest	18.2%	23.2%		15.0%	19.0%	
• Poorer	23.0%	21.1%		17.3%	20.0%	
• Middle	19.7%	19.1%		19.5%	20.2%	
• Richer	21.5%	18.5%		20.3%	21.0%	
• Richest	17.7%	18.0%		27.8%	19.8%	

Note: ^∗∗∗^*p* < 0.001

Based on the age group, women in the 40–44 age group occupied the multiparous Philippines and Indonesian women who use LTCM. On the other hand, the Philippines and multiparous Indonesian women who do not use LTCM are dominated by the younger age group, namely 35–39. According to the education level, the Philippines and multiparous Indonesian women with secondary education dominated the two categories of the use of LTCM.

Based on marital status, the Philippines and multiparous Indonesian women who are married/living with partners dominated the two categories of the use of LTCM. Meanwhile, based on employment status in both types, the use of LTCM is dominated by the Philippines and Indonesian employed women.

Finally, according to wealth status, multiparous Philippines women who use LTCM are dominated by the wealthy class with the lower category. On the other side, multiparous Philippines women who do not use LTCM are dominated by those with the most deficient wealth status category. Moreover, the wealthiest overwhelmed multiparous Indonesian women who use LTCM. On the other hand, the more decadent wealthy status category dominated multiparous Indonesian women who do not use LTCM.

### Multivariate analysis

Table 3 showed binary logistic regression results. In this final stage, the study involved all independent variables. The binary logistic regression uses “The use of LTCM: Yes” as references.

**Table 3:** The result of binary logistic regression of barriers for multiparous women to using LTCM in Philippines (*n* = 12,085) and Indonesia (*n* = 25,543)

Variables	Don’t use LTCM					
	Philippines			Indonesia		
	AOR	95% CI		AOR	95% CI	
		LB	UB		LB	UB
Place of residence: Urban	–	–	–	–	–	–
Place of residence: Rural	***1.070	1.070	1.070	***1.061	1.061	1.062
Age group: 15–19	***1.715	1.714	1.717	***2.360	2.356	2.365
Age group: 20–24	***1.467	1.466	1.467	***1.455	1.455	1.455
Age group: 25–29	***1.614	1.614	1.614	***1.231	1.231	1.232
Age group: 30–34	***1.317	1.317	1.317	***1.189	1.189	1.189
Age group: 35–39	***1.108	1.108	1.108	***0.900	0.900	0.901
Age group: 40–44	***0.806	0.806	0.806	***0.740	0.740	0.740
Age group: 45–49	–	–	–	–	–	–
Education: No education	***1.778	1.777	1.779	***3.966	3.964	3.967
Education: Primary	***0.951	0.950	0.951	***2.141	2.141	2.142
Education: Secondary	***0.800	0.800	0.801	***1.716	1.716	1.716
Education: Higher	–	–	–	–	–	–
Marital: Single	***1.699	1.699	1.699	***4.258	4.256	4.259
Marital: Married/Living with partner	–	–	–	–	–	–
Employment: Unemployed	***1.084	1.084	1.084	***1.044	1.044	1.044
Employment: Employed	–	–	–	–	–	–
Wealth: Poorest	***1.155	1.155	1.155	***1.134	1.134	1.134
Wealth: Poorer	***0.895	0.894	0.895	***1.148	1.148	1.148
Wealth: Middle	***0.960	0.959	0.960	***1.094	1.094	1.095
Wealth: Richer	***0.883	0.883	0.883	***1.172	1.172	1.172
Wealth: Richest	–	–	–	–	–	–

Note: ^∗^*p* < 0.05; ^∗∗^*p* < 0.01; ^∗∗∗^*p* < 0.001

Table 3 showed that multiparous Philippines women living in rural areas are more likely to live in urban areas 1.070 times not to use LTCM (AOR 1.070; 95% CI 1.070–1.070). Meanwhile, multiparous Indonesian women living in rural areas were 1061 times more likely than those residing in urban areas not to use LTCM (AOR 1.061; 95% CI 1.061–1.062). This analysis indicates that living in rural areas is a risk factor for multiparous women in the Philippines and Indonesia for not using LTCM.

According to the age group, Philippines multiparous women in the age group 35–39 and under have a higher likelihood of not using LTCM than the 45–49 age group. Philippines multiparous women in the 40–44 age group are less likely to use LTCM than the 45–49 age group. Meanwhile, multiparous Indonesian women with the age group 30–34 and under have a higher likelihood of not using LTCM than the 45–49 age group. Multiparous Indonesian women in the age group over 35–39 and above are less likely to use LTCM. This information shows that younger multiparous women in the Philippines and Indonesia have a higher risk of not using LTCM.

Based on the education level, multiparous Philippines women with no education have a higher chance of not using LTCM than those with higher education. Multiparous Philippines women with primary and secondary education have a lower possibility of not using LTCM than those with higher education. On the other hand, multiparous Indonesian women with any education level have a higher probability of not using LTCM than those with higher education. This analysis indicates that inadequate education is a risk factor for multiparous women in the Philippines and Indonesia for not using LTCM.

According to marital status, the Philippines and multiparous Indonesian women with a single group (never in a union, widowed, divorced) have a higher probability than those with status married/living with partners not to use LTCM. The result shows that not having a partner is a risk factor for multiparous women in the Philippines and Indonesia for not using LTCM.

On the other side, unemployed multiparous women in the Philippines and Indonesia have a higher probability of not using LTCM than those employed. This analysis shows that the unemployed are a risk factor for multiparous women in the Philippines and Indonesia for not using LTCM.

Finally, based on wealth status, the poorest Philippines multiparous women have a higher likelihood of not using LTCM than the richest one. Multiparous Philippines women with other wealthy groups were less likely not to use LTCM than the richest. On the other hand, multiparous Indonesian women with any wealthy status have a higher chance of not using LARC than the richest. This analysis indicated that low wealth status has a higher probability than the richest multiparous in the Philippines and Indonesia not to use LTCM.

## Discussion

The study results found that living in rural areas was a risk factor for multiparous women in the Philippines and Indonesia for not using LTCM. Empirically, rural areas are often left behind compared to urban areas in development in all fields, including the health sector [28]. This situation impacts the availability of information and services to access LTCM for multiparous women [29, 30].

The study found that younger multiparous women in the Philippines and Indonesia had a higher risk of not using LTCM. This finding is in line with studies in India [31]. This condition is likely because older multiparous women tend to want to stop their reproductive period. Meanwhile, those who are young still have the desire to have more children [32, 33].

The research found low education to be a risk factor for multiparous women in the Philippines and Indonesia for not using LTCM. Women with better education levels better understand their needs. Moreover, with better education, women also better understand the risk factors for any action or decision they take [34, 35], including better understanding the myths circulating about LTCM [36]. This reason is why multiparous women with higher education prefer to use LTCM [37–39]. Higher education levels have been found in several previous studies to positively determine health performance [38]. Conversely, several studies found poor education to be a barrier to higher-quality implementation in the health sector [40–42].

On the other hand, not having a partner was a risk factor for multiparous women in the Philippines and Indonesia for not using LTCM. In the context of countries with Eastern customs such as the Philippines and Indonesia, sexual activity for women who do not have a partner is a disgrace [43, 44]. This condition encourages women to control their sexual activity. This situation is likely to suppress the need for LTCM in unmarried multiparous women.

Meanwhile, the unemployed was a risk factor for multiparous women in the Philippines and Indonesia for not using LTCM. This situation may be due to employed multiparous women who prefer practical and effective contraceptive methods [45, 46]. Previous studies have shown the higher effectiveness of LTCM than non-LTCM methods [47]. On the other hand, LTCM is more effective for employed multiparous women than the forgetfulness factor caused by being busy. Other study revealed that women empowerment factors, one of it is employment, related to the utilization of LTCM [13].

Finally, the study found low wealth status to have a higher probability than the richest multiparous in Indonesia not to use LTCM. The previous studies saw poverty as a barrier to performance output in many health sectors, especially those related to and requiring costs [27, 48]. This situation also applies to poor multiparous women’s access to LTCM [49, 50]. This cost is a barrier that should encourage the government to realize the financing mechanism through insurance for LTCM [38, 51, 52].

In summary, this research shows that six variables prevent a multiparous woman from using LTCM, namely: living in the rural area, younger age, poor education, single, unemployed and poor. The implication of this situation for a country is an uncontrolled population explosion, leading to repeated unfavorable conditions in society, including poverty, ignorance, and high unemployment [53]. Another negative situation is the risk of an unqualified future generation because their parents cannot optimally carry out child care [54].

Previous research has recommended strategies to increase the use of LTCM, including reducing costs to get LTCM services, increasing public understanding of LTCM [55], and increase the skill capacity of health workers [56]. We can develop a strategy by cooperating with relevant stakeholders to make the LTCM program a success in an area [57]. From the findings above, appropriate policy recommendations include: providing subsidies for LTCM services for the poor, initiating an insurance model for LTCM, educating the public massively, being innovative, creative, and measurable, and periodically updating the skills capacity of health workers.

### Study limitation

This study has a limitation: the study’s variable is limited and depends on the availability of secondary data (DHS data). However, the study has a positive impact on the maternal and child health programs in Indonesia, such as this study can be estimated to the national level with correct weight to describe the level of the problem at the national level. Furthermore this study can be used by the government to develop policy based on the scientific evidence, especially related to LCTM and how the strategy of the government to deliver it to the specific population in the country.

## Conclusions

The research results concluded that there were six barriers for multiparous women to using LTCM in the Philippines and Indonesia. The six obstacles are living in rural areas, being younger, poor education, single, unemployed, and low wealth. Based on the findings of the research, the author recommend to the Philippines and Indonesia government to integrate the public health program and family planning program that can addres to the rural areas, teenagers, low education, unemployed group and poor people.

## Data Availability

The authors cannot publicly share the data because a third party and authors do not have permission to share it. The 2017 IDHS data set name requested from the ICF ‘data set of childbearing age women’ is available from the ICF contact https://dhsprogram.com/data/new-userregistration.cfm for researchers who meet the criteria for access to confidential data.

## Abbreviations

LTCM: Long-term contraceptive methods
IUD: Intrauterine device
PDHS: Philippines Demographic and Health Survey
IDHS: Indonesian Demographic and Health Survey
ICF: Inner City Fund
VIF: Variance inflation factor
AOR: Adjusted odds ratio
